# Screening with an NMNAT2-MSD platform identifies small molecules that modulate NMNAT2 levels in cortical neurons

**DOI:** 10.1038/srep43846

**Published:** 2017-03-07

**Authors:** Yousuf O. Ali, Gillian Bradley, Hui-Chen Lu

**Affiliations:** 1 Linda and Jack Gill Center, Department of Psychological and Brain Sciences, Indiana University, Bloomington, Indiana, United States of America; 2 The Cain Foundation Laboratories, Texas Children’s Hospital, Houston, Texas, United States of America; 3 Jan and Dan Duncan Neurological Research Institute, Texas Children’s Hospital, Houston, Texas, United States of America; 4 Department of Pediatrics, Baylor College of Medicine, Houston, Texas, United States of America; 5 Developmental Biology Program and Department of Neuroscience, Baylor College of Medicine, Houston, Texas, United States of America

## Abstract

Nicotinamide mononucleotide adenylyl transferase 2 (NMNAT2) is a key neuronal maintenance factor and provides potent neuroprotection in numerous preclinical models of neurological disorders. NMNAT2 is significantly reduced in Alzheimer’s, Huntington’s, Parkinson’s diseases. Here we developed a Meso Scale Discovery (MSD)-based screening platform to quantify endogenous NMNAT2 in cortical neurons. The high sensitivity and large dynamic range of this NMNAT2-MSD platform allowed us to screen the Sigma LOPAC library consisting of 1280 compounds. This library had a 2.89% hit rate, with 24 NMNAT2 positive and 13 negative modulators identified. Western analysis was conducted to validate and determine the dose-dependency of identified modulators. Caffeine, one identified NMNAT2 positive-modulator, when systemically administered restored NMNAT2 expression in rTg4510 tauopathy mice to normal levels. We confirmed in a cell culture model that four selected positive-modulators exerted NMNAT2-specific neuroprotection against vincristine-induced cell death while four selected NMNAT2 negative modulators reduced neuronal viability in an NMNAT2-dependent manner. Many of the identified NMNAT2 positive modulators are predicted to increase cAMP concentration, suggesting that neuronal NMNAT2 levels are tightly regulated by cAMP signaling. Taken together, our findings indicate that the NMNAT2-MSD platform provides a sensitive phenotypic screen to detect NMNAT2 in neurons.

Optimal brain function requires that neurons respond appropriately to a range of environmental challenges. Robust protective mechanisms in neurons protect and minimize damage arising from various stress and insults. Recent studies have revealed a key neuronal maintenance and protective function for nicotinamide mononucleotide adenylyl transferases (NMNATs)^1^,^2^. NMNATs can act as enzymes synthesizing nicotinamide adenine dinucleotide (NAD)^3^,^4^, an essential cofactor in many cellular processes^5^ as well as molecular chaperones^6^,^7^. Overexpressing NMNATs provides neuroprotection in several neurodegenerative conditions^6^,^7^,^8^,^9^,^10^,^11^,^12^,^13^,^14^,^15^,^16^,^17^,^18^,^19^.

Among the three NMNATs, NMNAT2 has the shortest half-life and is required for axonal survival^14^,^20^. NMNAT2 is also the major NMNAT in mammalian brains^21^,^22^. Reducing NMNAT2 levels in superior cervical ganglia neurons causes axonal degeneration^20^ and NMNAT2 null mutant mice exhibit axonal outgrowth deficits^20^,^23^. Supporting its role as a potential neuronal/axonal maintenance factor, *Nmnat2* mRNA expression is reduced in many neurodegenerative diseases^24^,^25^,^26^,^27^,^28^,^29^. In rTg4510 transgenic mice, a Frontotemporal Dementia and Parkinsonism-17 (FTDP-17) tauopathy model, NMNAT2 abundance declines prior to the onset of neurodegeneration or memory deficits^13^. Most importantly, NMNAT2 over-expression protects against hippocampal neuronal loss in rTg4510 mice.

Our previous studies found NMNAT2 levels in Alzheimer’s disease (AD) brains are about 30% of control brains^6^. Interestingly, NMNAT2 levels in the brains from Non-Demented with Alzheimer’s disease Neuropathology subjects (NDAN; possessing the same levels of plaque and tangle burden as the AD brains; ref. 30) are about 60% of control levels. In addition, *Nmnat2* mRNA levels positively correlate to human cognitive function while correlating negatively to AD related pathology^6^. Hence, it is important to elucidate the signaling pathways that are involved in regulating NMNAT2 levels.

To identify small molecules that can modulate NMNAT2 levels, we developed a high-throughput screening platform to detect endogenous NMNAT2 levels in cortical neurons with high fidelity and great dynamic range. The Sigma LOPAC library, with 1280 compounds that represent a total of 50 distinct mechanism classes^31^,^32^, was chosen to identify NMNAT2 modulators. Two secondary screens were conducted to identify potent NMNAT2-selective modulators. The nature of the small molecules identified to regulate NMNAT2 abundance suggests several upstream pathways modulate NMNAT2 levels in cortical neurons. We believe the knowledge of these pathways may promote better translational approaches for targeting NMNAT2 in various neurodegenerative diseases.

## Results

### Designing an NMNAT2-specific high-throughput screening (HTS) platform to quantify NMNAT2 protein levels in cortical neurons

The Meso Scale Discovery (MSD) detection platform potentially provides superb sensitivity with great dynamic range for detecting analytes of choice (MULTI-ARRAY^®^ technology and see http://www.meso-scale.com/ for details)^33^,^34^. This proprietary SULFO-TAG labeling allows for emission of light upon electrochemical stimulation initiated at the electrode surfaces of multi-array plates. The decoupling of stimulation and signal leads to strong and specific MSD signals. MSD relies on an ELISA sandwich assay that utilizes a “capture” antibody to bind the target protein in analyte. This Ab-Analyte complex is then recognized by a sulfo-tagged primary antibody (detection antibody) allowing detection. Here we aimed to design an NMNAT2-MSD platform utilizing commercially available NMNAT2 antibodies to reliably measure NMNAT2 levels.

**Figure 1:**
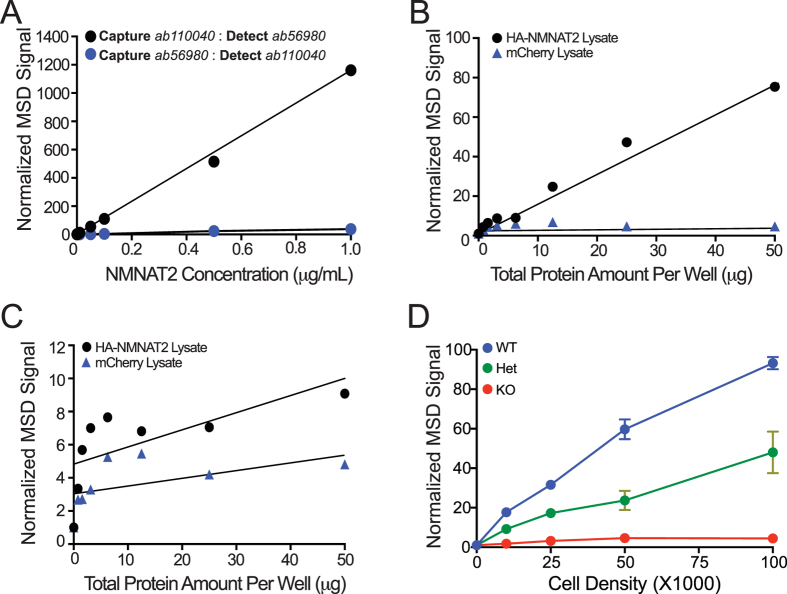
Development of an NMNAT2-MSD platform specifically detecting NMNAT2 with high sensitivity. (**A**) Ab110040 antibody as capture and sulfotagged Ab56980 antibody as the detection antibody pair, but not in reverse order provides, a strong NMNAT2-MSD signal and a linear detection with increasing concentrations of recombinant NMNAT2 protein. (**B**) Dose-dependent MSD signals for cell lysates of Cos-7 cells transfected with HA-NMNAT2 or mCherry. (**C**) Low NMNAT2-MSD signals for Cos-7 cells transfected with HA-NMNAT2 or mCherry when the order of capture and detection antibody was reversed. (**D**) NMNAT2-MSD signals detected from DIV 14 NMNAT2 WT, HET and KO neurons plated at the indicated densities.

A variety of commercially available monoclonal and polyclonal antibodies targeting either mouse or human NMNAT2 protein were acquired (Table S1). To start the optimization process, we designed the NMNAT2-MSD assay with different combinations of antibodies. Our first goal was to detect NMNAT2 protein in a dose-dependent manner with strong and specific signals (Fig. 1A). Different combinations of capture and detection antibodies were assembled and their detection efficacies were evaluated. We found that the best pair of antibodies providing the desirable linear dynamic range of detection was to use the Abcam ab110040 rabbit polyclonal antibody (epitope is aa100–200 of Rat NMNAT2) as the capture antibody together with sulfotagged-Abcam ab56980 mouse monoclonal antibody (epitope is aa208–308 of human NMNAT2) as the detection antibody (Fig. 1A). The signals were greatly reduced when the antibody order was reversed (Fig. 1A). This suggests that the proper combination of capture and detection antibody allowing for optimal conformational coverage of the analyte protein is important. In addition, almost no signal was detected when NMNAT1 protein was used as analyte (Fig. S1A) or when NeuN antibody was used as the capture antibody (Fig. S1B). At the same time, the signals were saturated when a very high quantity of recombinant NMNAT2 was placed as analyte (Fig. S1C). These finding from control experiments demonstrated that NMNAT2-MSD platform established here provides sensitive NMNAT2-specific detection.

To test if we could detect NMNAT2 in cultured cells, cDNAs carrying HA-tagged NMNAT2 or mCherry (as a negative control) were transfected into cos7 cells (Fig. 1B). The signal increased in a linear fashion in proportion to the total amount of protein prepared from NMNAT2-cos7 cells (Fig. 1B), while minimal signal was detected with mCherry-cos7 cell extracts. Reversing the capture and detection antibody order, again, greatly reduced the signals despite large amounts of total protein were applied (Fig. 1C).

**Figure 2:**
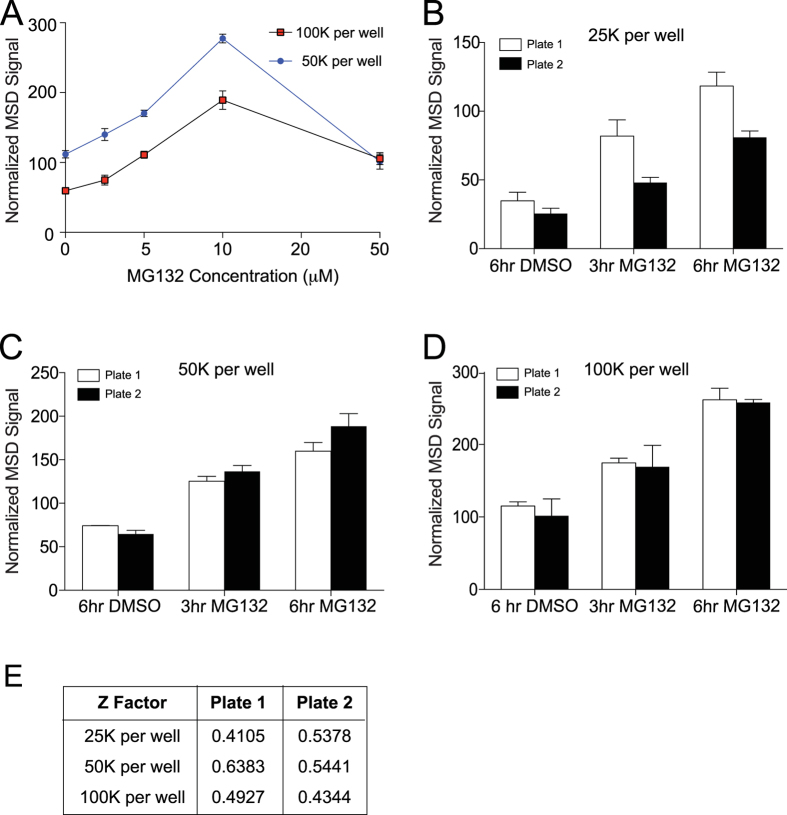
The NMNAT2-MSD platform detects dynamic changes in NMNAT2 protein levels in primary cortical neuron cultures in specific manner. (**A**) NMNAT2-MSD signals from cortical neurons treated with the indicated concentrations of MG132. (**B**–**D**) NMNAT2-MSD signals for neurons treated with 3 or 6 hrs of MG132. Two separate plates were examined. Neurons were plated at 25,000 (**B**), 50,000 (**C**), or 100,000 (**D**) cells per well of 96 well plates. (**E**) Summary of z factors for MG132-enhancement of NMNAT2 levels as measured by MSD.

NMNAT2 is expressed in cultured cortical neurons and its abundance is greatly increased between 8 and 13 days-*in-vitro* (DIV)^21^. NMNAT2 knockout (KO) mice die at birth^23^ but one can prepare NMNAT2 KO cortical neurons from NMNAT2 KO embryos. These neurons survive relatively well for more than two weeks *in vitro*^6^. To test if MSD detects endogenous NMNAT2, we prepared cortical neurons from E16.5 NMNAT2-wildtype (WT), heterozygous (HET) and KO embryos harvested from pregnant NMNAT2-HET dams mated with NMNAT2-HET male mice. These neurons were plated on poly-D-lysine–coated 96 well plates with ~12,500, 25,000, 50,000 or 100,000 cortical neurons per well. The signal to background ratio values from *Nmnat2* WT and HET DIV14 neurons linearly increased proportional to neuronal density (Fig. 1D). Most importantly, only minimal signal was detected with NMANT2 KO neurons while NMNAT2 MSD signal from *Nmnat2* HET DIV 14 neurons are ~50% of WT neurons. In addition, treatment with different concentrations of MG132, a proteasome inhibitor known to stabilize NMNAT2^14^,^20^,^35^, increased the NMNAT2 MSD signal in a dose-dependent fashion up to 10 μM (Fig. 2A). Together these results suggest that this NMNAT2-MSD assay format detects endogenous NMNAT2 in cortical neurons with great specificity and sensitivity.

Inter-plate variability was examined with densities of 25,000, 50,000 or 100,000 neurons per well. Neurons at these different densities were treated with vehicle or 10 uM MG132 for 3 or 6 hours (Fig. 2B–D). We found that 50,000 neurons per well gave the minimal inter-plate variability (Fig. 2B–D). The Z-factor is a standard measure for determining the ability of an assay to reliable detect a “hit” (see Methods). We found that neuronal densities of 50,000 cells per well gave Z factors >0.5 (Fig. 2E). Suggesting this cell density in 96 well plate will give the most reproducible signals while reducing the number of neurons needed in each assay.

Based on the manufacturer instructions (MSD.com), the optimal formulation and concentrations of antibodies for MSD assay should be 1–2 mg/ml of protein in PBS (pH 7.4–7.9), without any azide, carrier protein, glycine, histidine, Tris or glycerol (common additives in commercially available antibody solutions). Indeed, when the capture:detection antibody pair in optimal condition was acquired from Abcam, we found NMNAT2 MSD signals were increased for ~3.5 fold (Fig. S2). The large dynamic ranges of NMNAT2-specific MSD signals gave us confidence to conduct a drug screen using cortical neurons using the MSD technology.

### LOPAC library for Proof of Concept Screen

**Figure 3:**
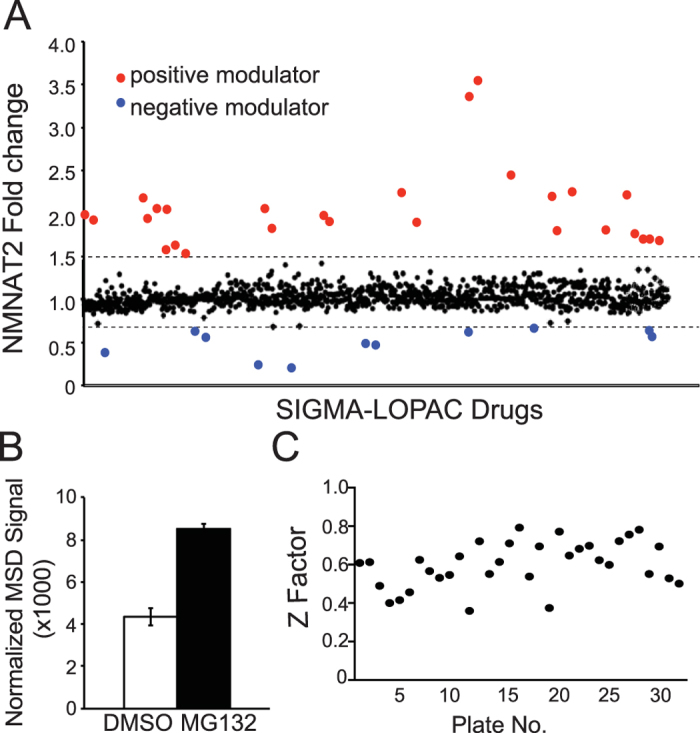
Identification of NMNAT2 modulators from the Sigma-LOPAC library screen using the NMNAT2-MSD platform. (**A**) NMNAT2 fold change from drug treatment of wild type cortical neurons plated in 96 well plates at density of 50 K per well and treated with 10 μM of the library compound for 6 hours. Fold change is represented as change over DMSO control. (**B**) Summary for NMNAT2-MSD signals detected from neurons treated with DMSO (negative control) and MG132 (positive control) from all the plates included in the data analysis. (**C**) Z factor distribution for all the assay plates included for data analysis.

To identify pathways modulating NMNAT2 abundance, we chose the SIGMA LOPAC library. The Sigma LOPAC^1280^ library contains 1280 pharmacologically-active compounds. This annotated collection of small molecule modulators and approved drugs impacts most cellular processes and covers all major drug target classes. Specifically, 10 uM of individual compounds from this library were applied to DIV14 cortical neurons plated at 50 K per well of 96 well plates (Fig. S3). Neurons in 96 well plates were treated by 10 uM of each compound in two separate plates, to account for interplate variability (Fig. S3). In every plate, 6 wells were treated with DMSO (negative control) and 6 wells were treated with 10 uM of MG132 (positive reference compound). After 6 hours of treatment, neurons were lysed with MSD buffer and prepared for NMNAT2-MSD assay. Z factors were calculated to examine inter-plate variability and only the results from a plate had Z-factor >0.4 were included for data analysis. NMNAT2-MSD signals were obtained from these treated neurons. We found that the normalized NMNAT2-MSD values of these compounds obtained were tightly clustered around a fold change of 1 (Fig. 3A), further demonstrating the reliability of the assay. Our positive reference compound MG132 yielded about 2-fold increase in NMNAT2 signals (Fig. 3B). Based on the distribution of Z factors, we found minimal inter-plate variability among different plates (Fig. 3C). The hit rate, defined by the number of drugs that either negatively or positively affected NMNAT2 abundance by 1.5 fold relative to DMSO treatment in this screen, was 2.89% (37 out of 1280) and resulted in identifying 24 positive and 13 negative NMNAT2 modulators (Table S2).

**Figure 4:**
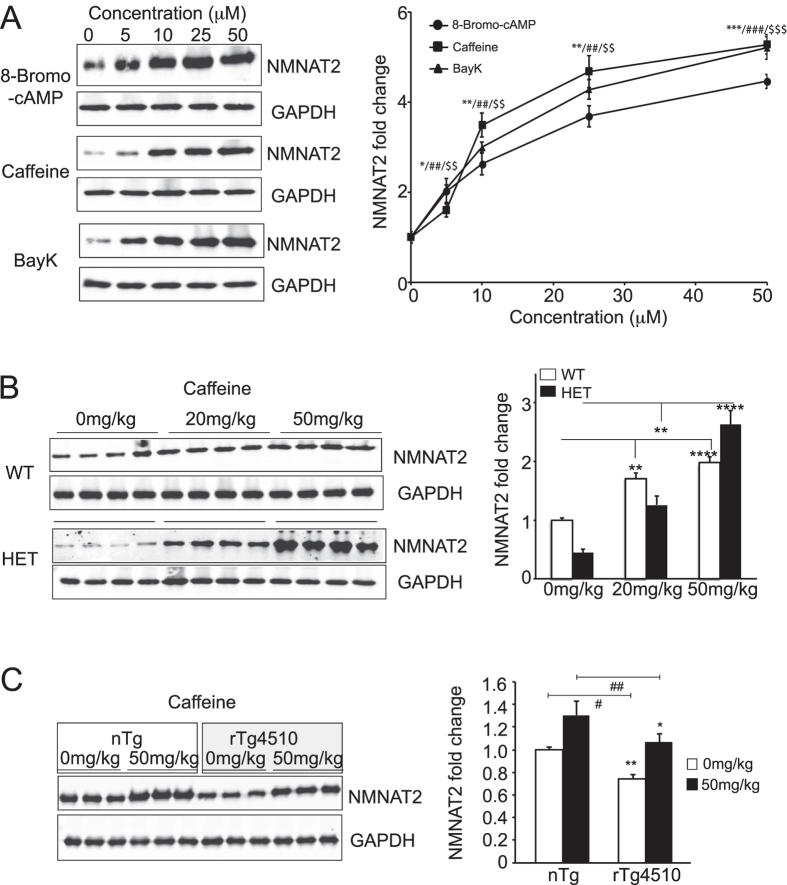
Caffeine increases NMNAT2 expression *in vitro* and in rTg4510 tauopathy mice. (**A**) Western blots show NMNAT2 and GAPDH levels in DIV14 cortical neurons after 6 hrs of treatment with 8-Bromo-cAMP, caffeine or Bay-K. Summary for the dose-dependent impact of 8-Bromo-cAMP, caffeine or Bay-K on NMNAT2 levels. *Caffeine/^#^BayK/^$^8-bromo-cAMP, ^*/#/$^p < 0.05, ^**/##/$$^p < 0.001, ^***/###/$$$^p < 0.0001 when compared to DMSO control for respective drugs. (**B**) NMNAT2 levels in the cortex of NMNAT2 WT and HET mice after saline or caffeine treatment (two independent experiments with n = 8 animals per treatment group). (**C**) NMNAT2 levels in the cortex of rTg4510 mice and controls after saline or caffeine treatment (repeated in two trials, with n = 6 total animals per treatment group). Bar graphs were plotted with mean ± sem; statistical significance was assessed by one-way ANOVA, *p < 0.05, **p < 0.001 (Complete original blots provided in Supplemental Figure S7).

Next, we conducted Western analysis with 33 hits (limited by compound availability and economical considerations) to validate and examine the potency of these hits. During the first round of testing, doses between 0–50 uM of 8-Br-cAMP, caffeine or Bay K were applied to DIV14 neurons for 6 hours to determine their impacts on NMNAT2 levels using the NMNAT2 2G8 antibody. Linear dose-dependent increases were found in the 0–20 uM range for all three compounds (Fig. 4A). Next, we examined the efficacy of 30 additional hits on NMNAT2 levels by treating neurons with DMSO/0, 1.25, 2.5, 5, 10 or 20 uM of the testing compound for 6 hrs (Figs S4 and S5). In total, 27 of 33 hits show similar degree of NMNAT2 level changes in MSD and Western (Table S2). Among these confirmed hits, 13 compounds induced significant changes in NMNAT2 level at the conc. of ≤2.5 uM (marked with yellow highlight on Table S2).

Impaired cell viability could account for the reduced NMNAT2 MSD signals. The MTT assay, a cell viability assay, was conducted to estimate the impacts of NMNAT2 negative modulators on the viability of DIV14 cortical neurons. We found that 6 hrs treatment of aconitine, cantharidin, E64, bendamustine HCL, wortmannin, Ziprasidone or retinoic Acid did not affect neuronal viability (Fig. S6). Etoposide and gossypol treatment did reduce neuronal viability.

Caffeine is commonly encountered in multiple beverages, has been linked to reduced dementia risk^36^, and is known to cross the blood brain barrier and thus we chose it to evaluate its *in vivo* impacts as a NMNAT2 positive modulator^37^. To examine whether caffeine could increase NMNAT2 in the brain, 2 different doses of caffeine (20 mg/kg or 50 mg/kg) or 0.9% saline vehicle were administered i.p. twice, separated by 4 hours, to 3-month-old NMNAT2 WT and HET mice. NMNAT2 levels in the cortex of these mice were evaluated 4 hours after the second dose with Western analysis. Similar to our previous finding, we found that NMNAT2 levels in NMNAT2 HET cortex was about 50% of the WT cortex (after vehicle treatment; Fig. 4B). Caffeine treatment resulted in a dose-dependent increase in NMNAT2 levels for both NMNAT2 WT and HET cortices. These data demonstrate that acute caffeine treatment can enhance NMNAT2 levels *in vivo* (Fig. 4B). Our previous studies found that NMNAT2 was greatly reduced in the cortex of rTg4510 transgenic mice, a tauopathy model^13^. To examine whether caffeine treatment could also increase NMNAT2 levels in the cortex of rTg4510 mice, 3-month-old rTg4510 mice and littermate controls were injected with vehicle or 50 mg/kg caffeine twice separated by 4 hours (n = 3 for each treatment). We found that cortical NMNAT2 levels were significantly increased in both control and rTg4510 mice after 8 hours of caffeine exposure (Fig. 4C).

### Vincristine-induced neuronal death is reduced by NMNAT2 positive modulators and enhanced by NMNAT2 negative modulators

**Figure 5:**
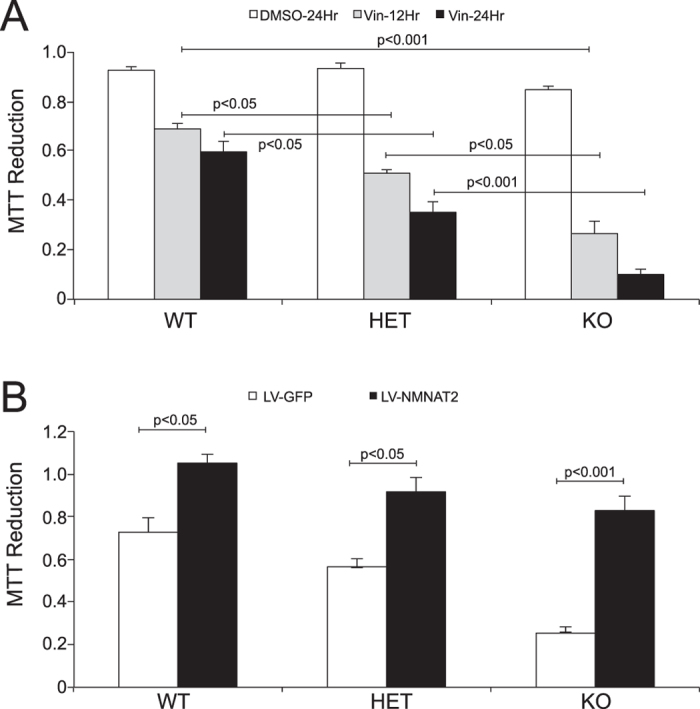
NMNAT2 protects against vincristine induced cell death. MTT assay shows reduced viability of NMNAT2 HET and KO neurons after vincristine exposure. (**A**) DIV14 NMNAT2 WT, HET, KO neurons were treated with DMSO or 10 uM vincristine for 12 or 24 hours. Cell viability was assessed with a MTT reduction assay. Experiment was repeated 3 times in triplicates. (**B**) NMNAT2 WT, HET, KO neurons were transduced with either LV-GFP or LV-NMNAT2 at DIV2. At DIV14, these neurons were treated with 10 uM vincristine for 24 hours. Experiment was repeated 3 times in triplicates. Statistical significance assessed using One-way ANOVA, followed by posthoc Tukey analysis.

Previously, we have demonstrated that NMNAT2 is required for neurons to counter the toxicity induced by vincristine, a chemotherapy agent commonly used to treat leukemia^38^. After 12 or 24 hours of vincristine treatment, the MTT assay was conducted to estimate cell viability of DIV14 NMNAT2 WT, HET and KO neurons. We found that vincristine treatment resulted in significant cell death in NMNAT2 WT neuron cultures by 12 hours of treatment (Fig. 5A). NMNAT2 HET and KO neurons were significantly more sensitive to vincristine than WT neurons. NMNAT2 KO neurons were particularly vulnerable to vincristine treatment. Less than of 10% of NMNAT2 KO neurons remained viable after 24 hours of vincristine treatment. Increasing NMNAT2 using a lentiviral vector in cultured cortical neurons offered significant protection against 12 hrs of vincristine-induced toxicity (Fig. 5B). Taken together with the dose-dependent correlation of NMNAT2 level to cell viability, this assay provides an approach to evaluate whether test compounds positively or negatively modulate NMNAT2-dependent neuroprotective effects.

**Figure 6:**
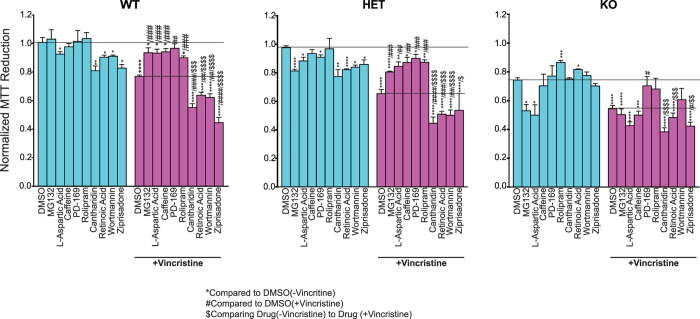
NMNAT2-specific neuroprotection assay. Cell viability was evaluated by MTT reduction. DIV14 NMNAT2 WT, HET, or KO neurons were pretreated with DMSO or 10 uM of the indicated compounds for 6 hours and then exposed to either DMSO (blue) or drug + vincristine (magenta) for additional 6 hours. Statistical significance within each group was assessed by two-tailed T-test, and intergroup differences were assessed by one-way ANOVA. *Compared to DMSO only; ^#^compared to DMSO + vincristine; ^$^compared to drug + DMSO. ^*/#/$^p < 0.05, ^**/##/$$^p < 0.001 ^***/###/$$$^p < 0.0001.

We next selected 4 positive NMNAT2 modulators: L-Aspartic acid, caffeine, PD169316, and Rolipram as well as 4 negative NMNAT2 modulators: cantharidin, Ziprasidone, retinoic acid, and wortmannin to evaluate whether they modulate sensitivity to vincristine-induced toxicity in an NMNAT2-dependent manner. DIV14 cortical neurons prepared from NMNAT2 WT, HET, or KO embryos were pretreated with 10 uM of the test compound for 6 hours first and then together with 10 uM vincristine or vehicle for an additional 6 hours. We found that pretreatment with L-Aspartic acid, caffeine, and rolipram significantly reduced the impact of vincristine on the cell viability of NMNAT2 WT and HET, but not KO neurons (Fig. 6), while PD-169316 pre-treatment reduced vincristine toxicity in all genotypes. These data suggest that L-Aspartic acid, caffeine, and rolipram require NMNAT2 to protect neurons from vincristine insult, while PD0169316 provides protection in an NMNAT2-independent fashion. NMNAT2-negative modulators: Ziprasidone, cantharidin, wortmannin and retinoic acid decreased cell viability in both NMNAT2 WT and HET but not KO neurons without vincristine. Upon vincristine treatment, these NMNAT2 negative modulators further reduced the viability of NMNAT2 WT and HET neurons. In NMNAT2 KO neurons, cantharidin, wortmannin and retinoic acid did not exaggerate vincristine-induced toxicity. Taken together these results suggest that 6 out of 8 NMNAT2 modulators affect neuronal viability by up regulating or down regulating NMNAT2 levels.

## Discussion

In this study, we show the NMNAT2-MSD-based assay developed here can detect NMNAT2 levels in cortical neurons with high specificity and great sensitivity. Using this assay, we screened 1280 pharmacologically active molecules from the Sigma LOPAC library and identified 37 NMNAT2 modulators. Twenty one modulators exerted a significant impact on NMNAT2 levels in the μM range. Systemic injection of caffeine, one of the identified NMNAT2 positive modulators, restored NMNAT2 expression to control levels in the rTg4510 mouse model of tauopathy. Notably, 6 out of 8 selected compounds modulated neuronal viability in an NMNAT2-specific manner. The nature of these compounds suggests that NMNAT2 levels can be upregulated via an increase of cAMP or excitatory neurotransmission. Taken together, our small molecule screen is the first to elucidate pathways regulating endogenous NMNAT2 levels. This information will help us to better understand the regulation of NMNAT2 levels in both healthy and diseased brains for future therapeutic development.

### Several pathways regulate NMNAT2 levels in cortical neurons

One of the advantages of using the Sigma-LOPAC library to screen is that its compounds are extensively annotated. This information allows us to form working hypotheses on which signaling cascades might modulate NMNAT2 levels. Among 17 NMNAT2 positive modulators confirmed by Western analysis, 5 compounds are known to increase cAMP signaling. These include 8-Br-cAMP, Ro20–1724^39^,^40^, caffeine^41^, the caffeine analog dipropyl-7-methylxanthine^42^, and rolipram^43^,^44^. Both caffeine and rolipram act as phosphodiesterase inhibitors to reduce cAMP degradation and thus increase cAMP concentrations. Interestingly, acute caffeine administration *in vivo* increased NMNAT2 levels more in NMNAT2-HET than in WT brains (Fig. 4B). Our previous study identified the presence of cAMP-response element binding protein (CREB) binding sites on the NMNAT2 promoter^13^. Indeed, we found that *nmnat2* mRNA levels in cortical neurons were significantly increased after 6 hrs of caffeine or rolipram treatments (Fig. S8). Chronic caffeine treatments have been examined in various neurodegenerative animal models. For example, Laurent *et al*. showed that chronic caffeine treatment in a tauopathy mouse model reduced tau hyperphosphorylation and improved memory function^45^. It remains to be determined whether caffeine induced increase in NMNAT2 levels account for the memory improvement seen in the Laurent study.

Many of the NMNAT2 positive modulators are known to enhance excitatory neurotransmission or be a consequence of enhanced neurotransmission, including: L-aspartic acid, Bay K8644, L-glutamic acid hydrochloride, pentylenetetrazole, quisqualic acid and thapsigargin. This suggests that neural activity level can also affect NMNAT2 abundance. It remains to be determined whether cAMP levels are affected by these positive modulators and then the increased cAMP augments NMNAT2 levels. In addition, two p38-alpha mitogen activated protein kinase (p38 MAPK) inhibitors were identified as NMNAT2 positive modulators. Recently, MAPK signaling was shown to promote NMNAT2 turnover, and inhibiting this signaling cascade increased NMNAT2 levels in *Drosophila* and mammalian motoneurons^46^. Interestingly, p38 MAPK activity has been associated with the hallmarks of AD and tau hyperphosphorylation^47^. Inhibitors of p38- α-MAPK have been shown to attenuate the inflammatory response and synaptic dysfunction seen in AD mouse models through unknown mechanisms^48^. It remains to be determined whether NMNAT2 is involved in this response.

Regarding NMNAT2 negative modulators, cantharidin potently inhibits protein phosphatase 2A (PP2A) activity in brain^49^,^50^, while wortmannin inhibits phosphatidylinositol 3-kinase (PI3-K). Ziprasidone HCl is an atypical antipsychotic drug that has been used to treat schizophrenia and bipolar disorder^51^. Interestingly, many NMNAT2 negative modulators have been used or developed as chemotherapy reagents to induce cell death (Table S2). These compounds include bendamustine hydrochloride, retinoic acid and PAC-1. Bendamustine hydrochloride causes cell cycle arrest through mitochondria dysfunction^52^. Some of these selected negative regulators (ziprisadone, wortmannin, cantharidin and retinoic acid) did not affect nmnat2 mRNA levels, suggesting their effects are mediated through different pathways, for example affecting NMNAT2 stability (Fig. S8).

Importantly, the vast majority of compounds in the LOPAC library exert little impact on NMNAT2 abundance. Thus (assuming the pathways are present in our system), the pathways where these compounds act are unlikely to be engaged in regulating NMNAT2 abundance. These compounds include: 89 compounds targeting dopamine signaling, 83 compounds acting on adrenoreceptor-mediated signaling, 68 compounds of the cholinergic pathway, and 68 compounds targeting serotonin signaling. Taken together, our screen identifies a limited number of pathways that can regulate NMNAT2 levels.

### NMNAT2-dependent neuroprotection against vincristine-induced cytotoxicity

Our current and previous studies show that reduction of NMNAT2 levels renders cortical neurons vulnerable to neural activity and vincristine toxicity^6^. Reducing NMNAT2 to 50% (NMNAT2 HET) or 0% (NMNAT2 KO) increases the vulnerability of cortical neurons to vincristine in a dose-dependent manner. Overexpressing NMNAT2 in NMNAT2 WT, HET, and KO neurons significantly increases their resistance to vincristine insult. These finding suggest that NMNAT2-dependent neuroprotection mechanism in cortical neurons is required to maintain neuronal health as well as to defend against vincristine insult.

Using this custom MTT-vincristine assay, we found that L-aspartic acid, caffeine, and rolipram pre-treatment significantly protected against vincristine toxicity as measured by cell viability and this neuroprotection requires NMNAT2. These compounds exert a modest impact on WT cortical neuron viability in the absence of vincristine. Pretreatment with PD-169316, a p38MAPK inhibitor, reduced vincristine toxicity in WT, HET and KO neurons. Thus inhibiting p38MAPK can offer NMNAT2-independent neuroprotection when NMNAT2 is reduced or loss.

Ziprasidone, cantharidin, wortmannin and retinoic acid are NMNAT2 negative modulators. As discussed above, these compounds reduce NMNAT2 levels without affecting neuronal viability after 6 hours. However, treatment with these compounds for 12 hours decreases cell viability in both WT and NMNAT2 HET cortical neurons. Interesting, these do not decrease viability in NMNAT2 KO neurons. This finding suggests that ziprasidone, cantharidin, wortmannin and retinoic acid reduce cell viability by decreasing NMNAT2 levels. Upon vincristine treatment, these NMNAT2 negative modulators further reduce the viability of WT and HET neurons. In summary, using an MTT-vincristine assay, we found that 6 out of 8 NMNAT2 modulators tested can affect neuronal viability in NMNAT2-dependent manner. Among these six compounds, only rolipram and caffeine, but not ziprasidone, cantharidin, wortmannin or retinoic acid, significantly impacted *nmnat2* mRNA levels (Fig. S8). These data suggest that the NMNAT2 negative modulators identified in this study reduce NMNAT2 levels post-transcriptionally and have minimal impact on *nmnat2* transcription.

In conclusion, we demonstrate that a variety of pharmacologically active compounds, and therefore multiple clusters of cellular pathways, are involved in the regulation of NMNAT2 abundance in cortical neurons. While increasing NMNAT2 abundance in neurodegenerative conditions may provide therapeutic benefit, it is equally if not more important to understand why NMNAT2 levels are reduced in various neurodegenerative conditions. We hypothesize that this risk could be associated with decreased NMNAT2 levels, which have already been shown to adversely affect human cognition^6^. In addition, retinoic acid is a derivative of retinoids/vitamin A. It will be very important to examine the relationship of NMNAT2 levels and the usage of ziprasidone and retinoids or vitamin A intake to further assess this relationship, especially in disease state or neurodegenerative conditions. Knowledge of NMNAT2 negative modulators provides useful insights into which pathways are naturally causing the decline of this necessary protein in disease conditions.

## Methods

### Animals

NMNAT2^blad^ (bloated bladder) heterozygotes were bred to generate wild type (NMNAT2^+/+^), heterozygous (NMNAT2^blad/+^) and knock-out (NMNAT2^blad/blad^) embryos^23^. rTg4510 mice were generated by F1 crossing of responder mice carrying tauP301L cDNA with an upstream tetracycline-operon responsive element, and activator transgenic lines, containing a trans-activator gene consisting of the tetracycline-off open reading frame placed downstream of the CaMKII promoter^53^,^54^. Genotyping procedure was conducted as described^23^,^53^,^54^. Animal care, housing and use were in compliance with the NIH Guidelines for the Care and Use of Laboratory Animals and were approved by the institutional animal care committee at Baylor College of Medicine and Indiana University.

### Chemical and Antibodies

All the antibodies and their sources are listed in Table S1. For NMNAT2 western blotting, 2G8 (Abnova) antibody was used. GAPDH (Sigma) was used for loading control. All drugs were obtained from Sigma.

### Primary Neuronal Culture

Heterozygous pregnant females were anesthetized according to an IACUC-approved protocol and embryos were extracted and rapidly decapitated. Cortical tissue from E16.5 ICR or NMNAT2 wildtype, NMNAT2 heterozygous or NMNAT2 knockout embryos was isolated. Cells were dissociated using the Worthington Papain Dissociation kit according to manufacturer’s protocol (Worthington Inc.). Genotyping was performed and analyzed during the dissociation process and cortices of identical genotypes were combined for plating. Neurons were plated at indicated densities, and 50,000 cells per well of a 96-well plate for LOPAC screening and MTT assays, or 500,000 cells per well of a 12-well plate, for Western Blotting. Cultures were maintained in a regularly replaced Neurobasal media (Invitrogen, Carlsbad, CA, USA) with B27 supplement (Invitrogen), penicillin-streptomycin supplement (Life Technologies) and L-glutamine (Invitrogen). Neurons were incubated at 37 degrees Celsius and 5% humidity for 14 days *in vitro* (DIV14) before being used in experiments.

### LOPAC Compounds Treatment

At DIV14, neuronal cultures were treated with specific compound of interest from the Library of Pharmacologically Active Compounds (LOPAC^®^1280) (Sigma-Aldrich, St. Louis, MO, USA) assortment. Allotments of all 1,280 compounds were provided by MD Anderson Cancer Center (Houston, TX, USA). Compounds were diluted to 10 uM in fresh Neurobasal media and dilutions were added directly to cultures for 6 hours of incubation. Neurons were lysed with Tris Lysis Buffer from MSD supplemented with Protease inhibitor and Phosphatase inhibitor tablets from Roche.

### MSD Platform Screen

Standard MSD 96-well plates were solution coated by adding 30 μL/well of 4 ug/mL ab11040 capture antibody in PBS. The plates were sealed and incubated overnight at 4 °C with no shaking. After the coating incubation, the plate was blotted dry on absorbent paper and the plate was then blocked using 150 μL/well of 5% Blocker A (MSD) in PBS for 1 hour at room temperature with shaking at 1000 rpm. The plate was then washed 3x for 10 minutes with PBS + 0.05% Tween-20 buffer. The NMNAT-2 calibrator (human NMNAT2 recombinant protein CF, R&D Systems, Catalog No. 6279-NT-010, supplied in PBS, pH 7.5 custom formulated for bulk order) in the dilutions indicated in the plate layouts were added to the plate (25 μL/well) in PBS/1% Blocker A on the top row of 96 well plate. Neuronal lysates were added at 150 uL to indicated wells and incubated at room temperature for 2 hours with shaking. The plate was then washed 3x with PBS + 0.05% Tween-20 buffer.

All detection MAbs, such as ab56980, were labeled using a challenge ratio of 20:1 of SULFO-TAG:target. Just before use, 150 nmole SULFO-TAG NHS ester was reconstituted with 50 μL H_2_O to prepare a 3 nmol/μ Lsolution.

For Abcam antibody (Ab-56980), with a concentration of 1 ug/mL:


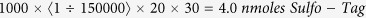


SulfoTAG stock solution was added to the protein solution incubated for 2 hours and then stored in the dark. 25 μL/well of anti-sulfotagged NMNAT-2 ab56980 at 1 μg/mL was then added to the plate, where indicated, in PBS/1% Blocker A and incubated at room temperature for 2 hours with shaking. The plate was washed 3x with PBS + 0.05% Tween-20 buffer. 150 μL/well of 1x read buffer was added and the plate was read immediately.

Since each of the experimental plates had positive (MG-132) and negative (DMSO) controls replicated in 6 separate wells, we calculated the Z factor as:


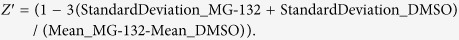


Z-factor^55^,^56^ was calculated from each plate and in general, we considered data from each plate for inclusion into our analysis if the Z-factor was above 0.4. MSD signals were subtracted with the average background signals from the wells with no neurons. Normalized MSD signals (fold change) for individual compounds were acquired by dividing the averaged MSD signals from wells containing neurons and drugs to the average MSD signals from the wells containing neurons with DMSO.

### Analysis of Cell Viability with MTT Assay

DIV14 neurons in individual wells of a 96 well plate were treated with 10 uM of the selected compound for 6 hours and then in combination with vehicle or 10 uM vincristine for an additional 12 hours. The Vybrant^®^ MTT Cell Proliferation Assay Kit (Life Technologies) was used to determine viable cells according to provided protocol. In brief, media was removed and replaced with 90 uL fresh Neurobasal media and 10 uL of 12 mM MTT (3-(4,5-dimethylthiazol-2-yl)-2,5-diphenyltetrazolium) in PBS solution. The cells were incubated for 4 hours. SDS-HCl solution was added directly to the wells, mixed and allowed to incubate for another 4 hours. Incorporated and reduced MTT levels of formazan were measured using an absorbance microplate reader at 576 nm (BMG Labtech Omega Plate Reader, BMG Labtech, Cary, NC). Three independent experiments with at least 5 replicate wells were performed for each treatment group.

### Immunoblotting

After treatment, primary cortical neurons (1 million per well) in 12-well plates were lysed with MSD-TRIS lysis buffer (MSD Inc.) supplemented with phosphatase and protease inhibitors (Life Technologies). Protein concentrations were measured according to previously described Bradford assay protocol. 10 ug protein in 10 uL was loaded per well of a 4–20% sodium dodecyl sulfate polyacrylamide precast gel (Bio-Rad, Hercules, California, USA). Protein was transferred to a nitrocellulose membrane (Bio-Rad). The membrane was incubated with NMNAT2 (2G8 clone), MAP2 (Cell Signaling, Danvers, MA, USA) or NeuN antibody (Cell Signaling) (all 1:1000) in a 1:4 blocking buffer (Rockland) dilution in TBS. After 3 washes with TBST (Tris-HCl, ph 7, 0.02% Tween 20), membranes were then incubated with the corresponding infrared dye conjugated secondary antibodies and images were acquired using an Odyssey CLx Infrared Imaging System (LI-COR, Lincoln, Nebraska, USA). Images were quantified with densitometry analysis with NIH ImageJ software.

### Real-time quantitative PCR (qPCR)

RNA was extracted from DIV14 neurons 6 hrs post treatment using the RNEASY Mini RNA Extraction kit (Qiagen), according to the manufacturer’s protocol. One μg of RNA per sample was reverse transcribed into cDNA using Superscript II (Invitrogen). Standard PCR amplification was performed using Sso Advanced Universal SYBRGreen Mastermix (BioRad) using the CFX Connect Real-Time PCR Detection System (Biorad, Hercules, CA). Primers used for detection of NMNAT2 were 5′-cagtgcgagagacctcatccc-3′ and 5′-acacatgatgagacggtgccg-3′ and for GAPDH were 5′-aaggtcatcccagagctgaa-3′ and 5′-ctgcttcaccaccttcttga-3′ (Sigma Aldrich, St. Louis, MO).

### Classification as Regulator of NMNAT2 Abundance

LOPAC compounds were classified as positive hits with MSD primary screen if the compound upregulated or downregulated NMNAT2 abundance by at least a 1.25 or −1.25 fold change, respectively. Compounds that did not correlate with a change in NMNAT2 abundance that reached or surpassed this threshold were characterized as misses. LOPAC compounds were classified as positive hits with western blotting if the compound upregulated or downregulated NMNAT2 abundance for at least half of the utilized concentrations by least a 1.25 or −1.25 fold change, respectively (Fig. S43). Therefore, if three of the five designated concentrations produced a 1.25 fold change, the compound was characterized as a NMNAT2 upregulator, regardless if the three concentrations were consecutive.

### Intraperitoneal Injections

Mice were injected (i.p.) with caffeine (dissolved in 0.9% sterile saline) at a dose of 20 mg/kg or 50 mg/kg twice, four hours apart. Brains were harvested four hours after the second injection and homogenized in Tris Lysis Buffer from MSD Inc. Similarly, 3-MO rTg4510 mice and control littermates were injected with 50 mg/kg of caffeine using the same paradigm above. Protein concentrations were measured using the Bradford method (BioRad).

### Statistical Analysis

Data of all MTT assays were assessed for statistical significance using GraphPad Prism7 software (GraphPad, San Diego, CA, USA). Summary data are represented as means +/− SEM. Means were compared between groups using one-way analysis of variance (ANOVA) for ≥ three groups or t-tests for two groups for data with normal distribution that met variance homogeneity. Significance was assessed using the Tukey criterion for pairwise mean comparisons under the ANOVA model.

## Additional Information

**How to cite this article**: Ali, Y. O. *et al*. Screening with an NMNAT2-MSD platform identifies small molecules that modulate NMNAT2 levels in cortical neurons. *Sci. Rep.* **7**, 43846; doi: 10.1038/srep43846 (2017).

**Publisher's note:** Springer Nature remains neutral with regard to jurisdictional claims in published maps and institutional affiliations.

## Supplementary Material

Supplementary Information
