# Corrigendum: Screening with an NMNAT2-MSD platform identifies small molecules that modulate NMNAT2 levels in cortical neurons

**DOI:** 10.1038/srep46780

**Published:** 2017-04-27

**Authors:** Yousuf O. Ali, Gillian Bradley, Hui-Chen Lu

Scientific Reports 7: Article number: 4384610.1038/srep43846; published online: 03 07 2017; updated: 04 27 2017

In the Supplementary Information file originally published with this Article, Supplementary Tables 1 and 2 were omitted. This error has been corrected in the Supplementary Information that now accompanies the Article.

