# Supplementary material for: Screening with an NMNAT2-MSD platform identifies small molecules that modulate NMNAT2 levels in cortical neurons
Source: Sci Rep. 2017 Mar 7;7:43846. doi: 10.1038/srep43846 (PMC5358788; doi:10.1038/srep43846)
Supplement: Supplementary Information [file srep43846-s1.pdf]

Supplementary Information

Title: Screening with an NMNAT2-MSD platform identifies small molecules that modulate NMNAT2 levels in cortical neurons.

Authors: Yousuf O. Ali[1-4*], Gillian Bradley[1,5], Hui-Chen Lu[1-5*]

Supplementary Figure Legends

Figure S1. NMNAT2-MSD platform detects NMNAT2 in specific manner. Three control experiments were conducted to demonstrate the specificity of this assay. A) Minimal MSD signals were acquired with increasing concentrations of NMNAT1 recombinant protein. B) Low MSD signals were acquired when NeuN antibody was used as capture antibody and Ab56980 antibody as detection antibody to detect NMNAT2 proteins. C) MSD signals reached plateau when a very high amount of NMNAT2 protein was used.

Figure S2. Optimization of Ab56890 and Ab110040 antibody formulation improves signal to noise ratio for detecting NMNAT2. The new antibodies were concentrated to 1mg/ml and were free of any additives.

Figure S3. Layout and plating scheme of LOPAC compounds for application into 96 well plate containing cultured neurons. (A) Layout of a 96-well plate treatment format for LOPAC drugs. Intermediate plates contained 100ul of 50uM LOPAC drugs to be applied in duplicate plates to account for interplate variability. 25ul of drug solutions were then added to neuronal cultures plated in the same format into 100ul Neurobasal media, yielding a final concentration of 10uM (Vt=125 ul). B) Scheme for making intermediate drug plates from Medchem plates (which contained original LOPAC drugs at 10mM concentration).

Figure S4. Western Analysis of the dose dependent effects of NMNAT2 positive modulators on endogenous neuronal NMNAT2 levels in WT cortical neurons. DIV14 WT neurons were treated with indicated concentrations of the drugs for 6 hours. The lysates were, separated by SDS-PAGE, transferred to nitrocellulose membranes, and probed for

NMNAT2 using the 2G8 antibody. NMNAT2 immuoreactivity was normalized to MAP2 signals (a neuronal marker). Levels of NMNAT2 were expressed as fold change relative to the DMSO control.

Figure S5. Western Analysis of the dose dependent effects of NMNAT2 negative modulators on endogenous neuronal NMNAT2 levels in WT cortical neurons. DIV14 WT neurons were treated with indicated concentration of the drug for 6 hours and analyzed as in **Figure S4**.

Figure S6. MTT assay to determine neuronal survival after a 6 hr treatment with various NMNAT2 negative modulators. Only etoposide and gossypol reduced cell viability following a 6 hr treatment at 10uM concentration.

Figure S7. Raw Blots for figure 4.

Figure S8. Quantitative RT-PCR to determine the impacts of drug treatment on *nmnat2* mRNA level. WT cortical neurons were treated with 10μM of each drug for 6 hrs and total RNA were extracted for qPCR experiments (please see Methods). *nmnat2* mRNA levels were normalized to GAPDH loading control and plotted as fold change to DMSO control. MG132 is a proteasome inhibitor. For MSD assay, it was used as a positive control to increase NMNAT2 protein level because it reduces the degradation of NMNAT2 by proteasome. Here we show that MG132 treatment has no impact on nmnat2 mRNA abundance. *$p<0.05$

Table S1. List of Commercial NMNAT2 antibodies tested in our study.

Table S2. List of 37 compounds identified by NMNAT2-MSD screen name, simplified descriptions of their actions, NMNAT2 fold changes detected by MSD, and NMNAT2 fold changes detected by Western analysis. Drugs that showed a consistent impact on NMNAT2 levels are labeled in blue, while compounds with inconsistent changes are labeled in grey. The rest are in black. Note the fold change shown is from drug treatment at 10uM concentration for 6 hours. The drug functional description is adapted from the LOPAC library annotations. Blue compounds that are highlighted in yellow identify those that significantly affected NMNAT2 levels at a concentration ≤ 2.5 μM.

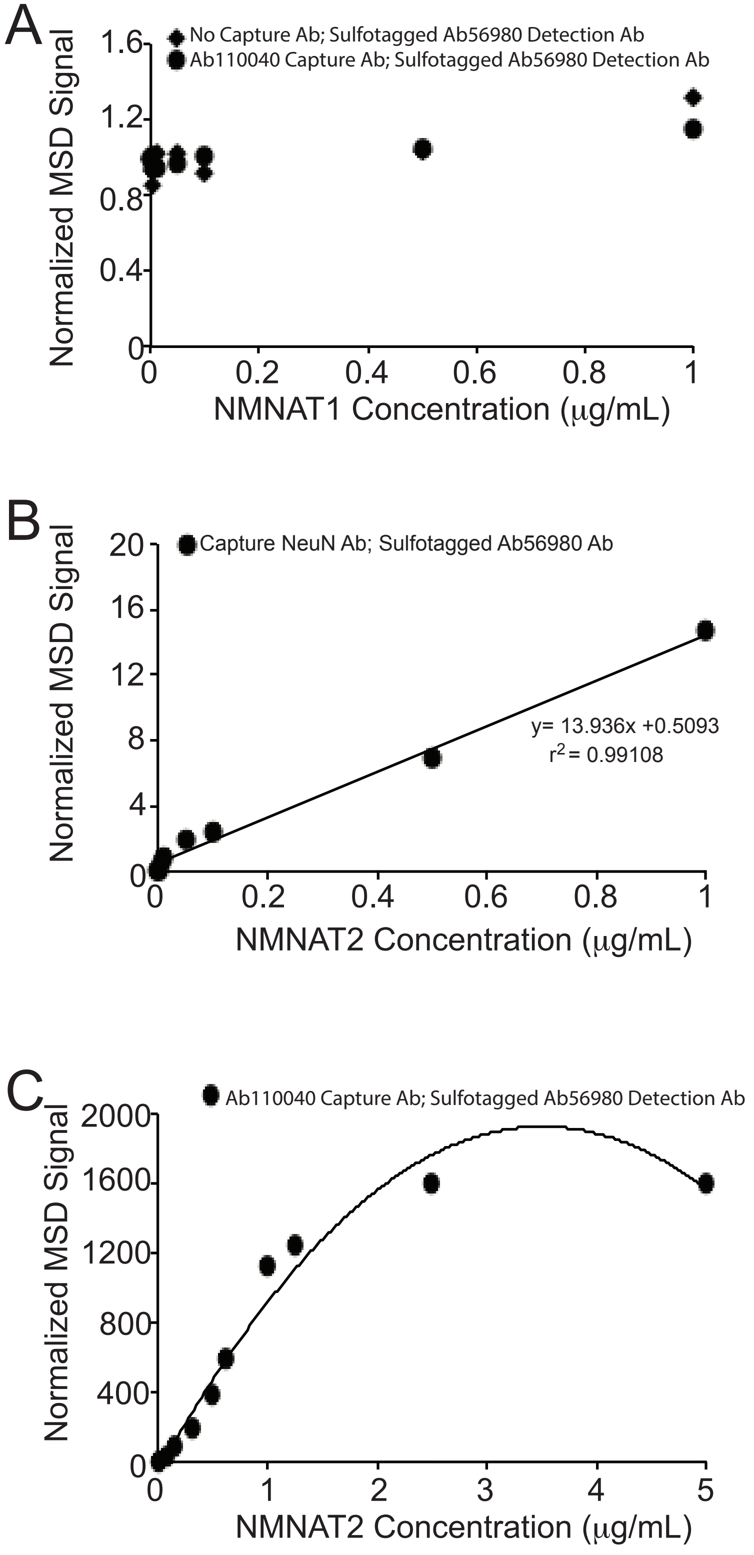
A
No Capture Ab; Sulfotagged Ab56980 Detection Ab
Ab110040 Capture Ab; Sulfotagged Ab56980 Detection Ab
Normalized MSD Signal
1.6
1.2
0.8
0.4
0
0
0.2
0.4
0.6
0.8
1
NMNAT1 Concentration (μg/mL)
B
Capture NeuN Ab; Sulfotagged Ab56980 Ab
Normalized MSD Signal
20
16
12
8
4
0
y= 13.936x +0.5093
r2= 0.99108
0
0.2
0.4
0.6
0.8
1
NMNAT2 Concentration (μg/mL)
C
Ab110040 Capture Ab; Sulfotagged Ab56980 Detection Ab
Normalized MSD Signal
2000
1600
1200
800
400
0
0
1
2
3
4
5
NMNAT2 Concentration (μg/mL)

Figure S1

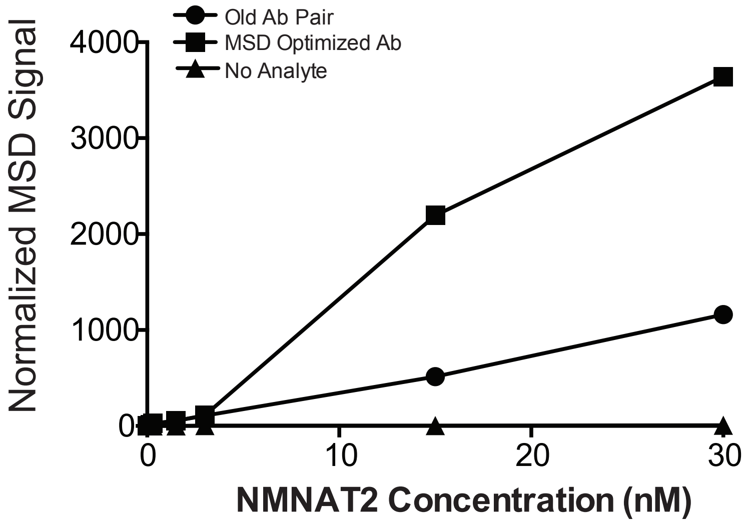

Old Ab Pair
MSD Optimized Ab
No Analyte
Normalized MSD Signal
4000
3000
2000
1000
0
0
10
20
30
NMNAT2 Concentration (nM)

Figure S2

A

	LOPAC Compounds								Neg. Control	Pos. Control	
XX	XX	XX	XX	XX	XX	XX	XX	XX	XX	XX	XX
XX	D1	D2	D3	D4	D5	D6	D7	D8	DMSO	MG132	XX
XX	D9	D10	D11	D12	D13	D14	D15	D16	DMSO	MG132	XX
XX	D17	D18	D19	D20	D21	D22	D23	D24	DMSO	MG132	XX
XX	D25	D26	D27	D28	D29	D30	D31	D32	DMSO	MG132	XX
XX	D33	D34	D35	D36	D37	D38	D39	D40	DMSO	MG132	XX
XX	D41	D42	D43	D44	D45	D46	D47	D48	DMSO	MG132	XX
XX	XX	XX	XX	XX	XX	XX	XX	XX	XX	XX	XX

B

Medchem plate preparation:

1. 20 ul of each compound from library's titer tubes were transferred into respective medchem plates.
2. Additionally, 20 uL of DMSO was added into each well, thus diluting compounds from 10mM to 5 mM as 40 uL offers better coverage of bottom of each well. This will ensure that robot tips will aspirate the expected amount.
3. DMSO was added into column 10.

Intermediate Plate Preparation:

1. 3 uL of each compound will be delivered into 297 ul of media and mixed, diluting 100-X further to 50 uM.
2. MG132 Intermediate Solution was added directly into column 11 of intermediate plate to be certain there is no precipitation problem.

Cell Plate Preparation:

1. 25 uL from each intermediate dilution from intermediate plate will be transferred into cells containing 100 uL media.
2. This will be the last dilution (5X) resulting in a final 10 uM compound concentration.

Figure S3

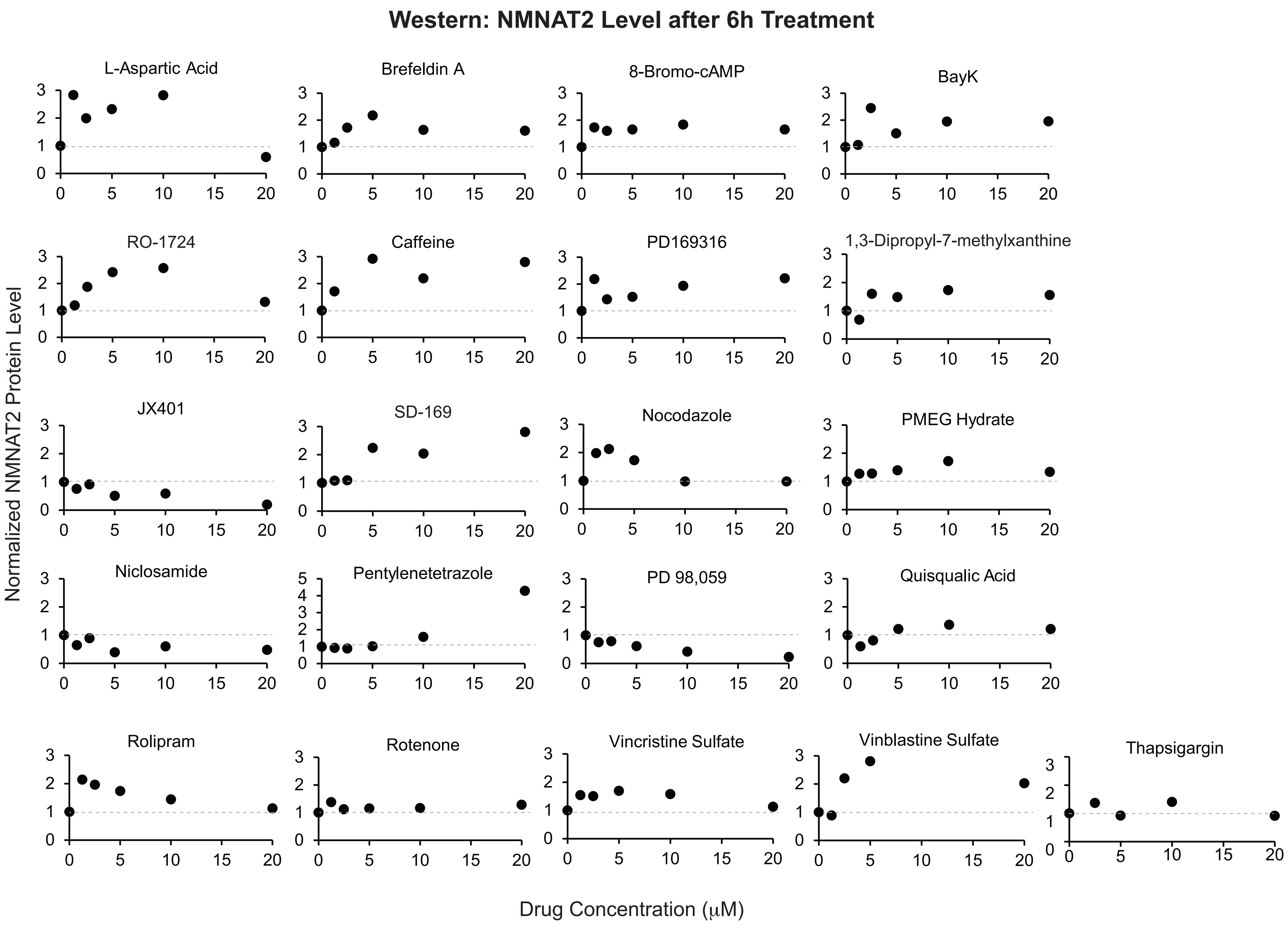
Western: NMNAT2 Level after 6h Treatment
L-Aspartic Acid
Brefeldin A
8-Bromo-cAMP
BayK
RO-1724
Caffeine
PD169316
1,3-Dipropyl-7-methylxanthine
JX401
SD-169
Nocodazole
PMEG Hydrate
Niclosamide
Pentylenetetrazole
PD 98,059
Quisqualic Acid
Rolipram
Rotenone
Vincristine Sulfate
Vinblastine Sulfate
Thapsigargin
Normalized NMNAT2 Protein Level
Drug Concentration (μM)

Figure S4

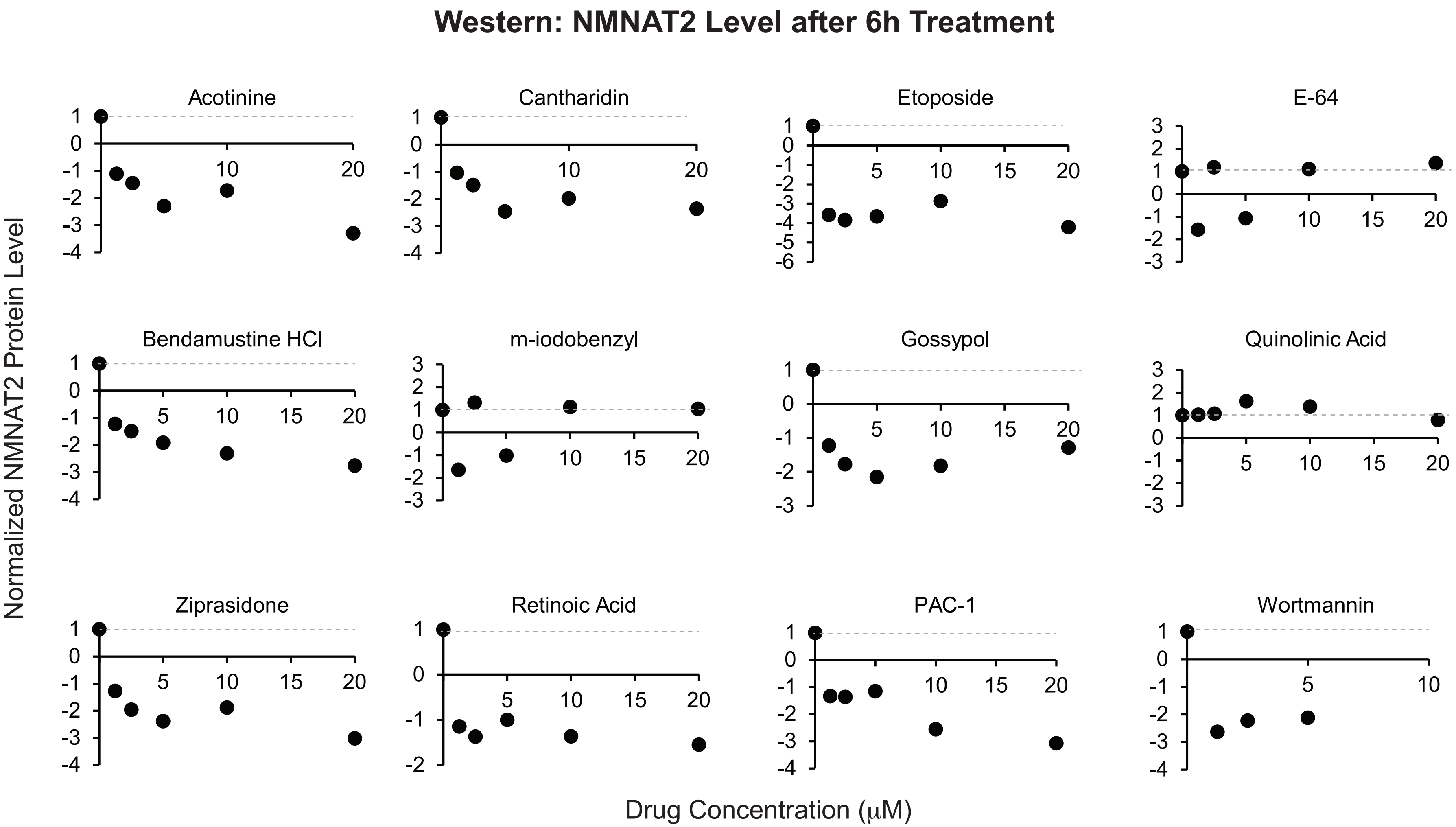

Western: NMNAT2 Level after 6h Treatment
Acotinine
Cantharidin
Etoposide
E-64
Bendamustine HCl
m-iodobenzyl
Gossypol
Quinolinic Acid
Ziprasidone
Retinoic Acid
PAC-1
Wortmannin
Normalized NMNAT2 Protein Level
Drug Concentration (μM)

Figure S5

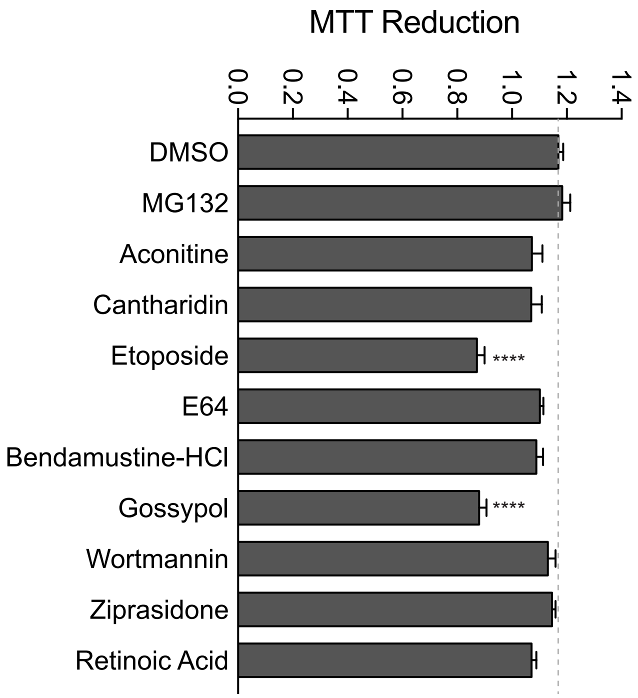
MTT Reduction
0.0
0.2
0.4
0.6
0.8
1.0
1.2
1.4
DMSO
MG132
Aconitine
Cantharidin
Etoposide
****
E64
Bendamustine-HCl
Gossypol
****
Wortmannin
Ziprasidone
Retinoic Acid

Figure S6

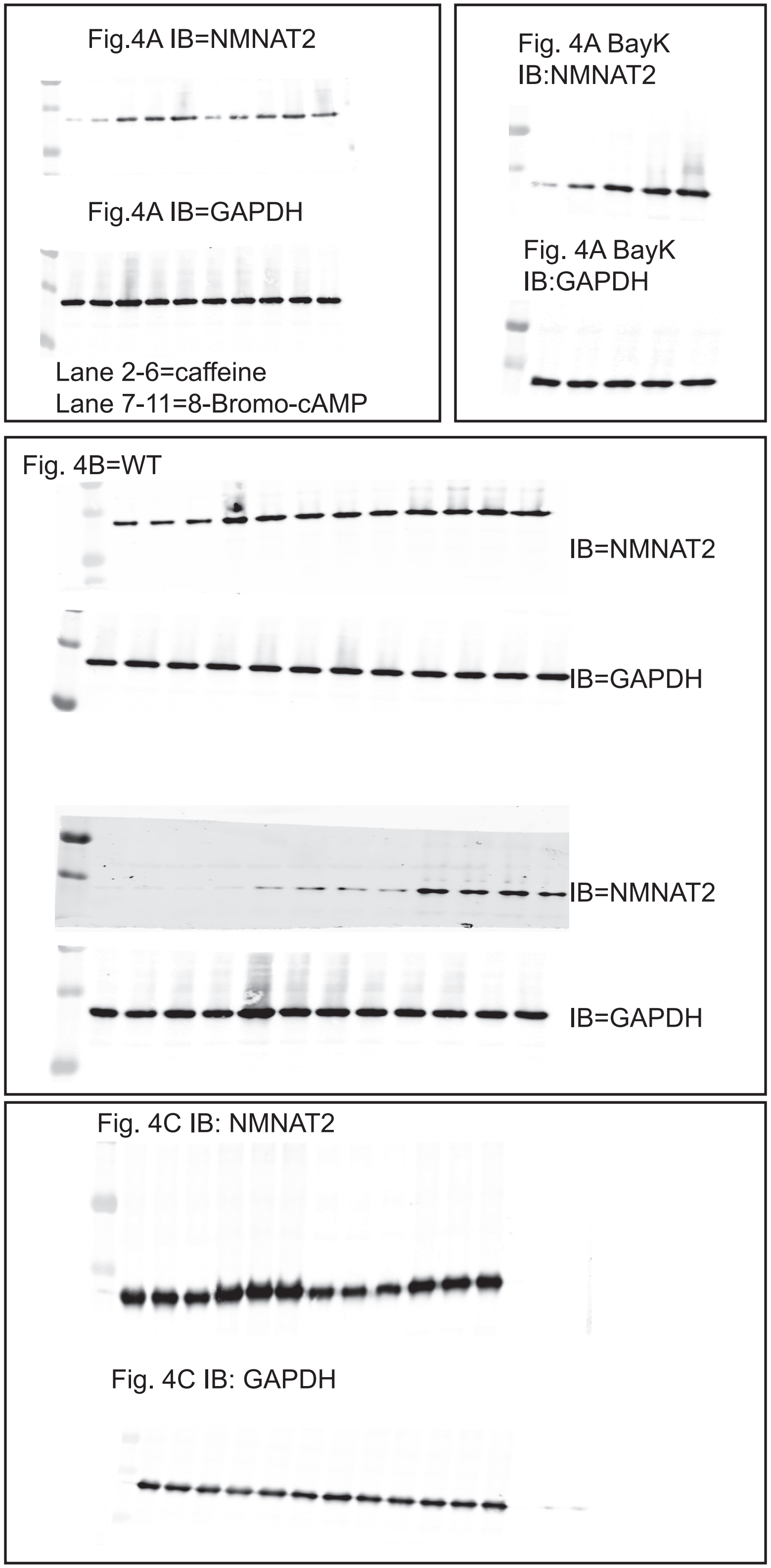
Fig.4A IB=NMNAT2
Fig.4A IB=GAPDH
Lane 2-6=caffeine
Lane 7-11=8-Bromo-cAMP
Fig. 4A BayK
IB:NMNAT2
Fig. 4A BayK
IB:GAPDH
Fig. 4B=WT
IB=NMNAT2
IB=GAPDH
IB=NMNAT2
IB=GAPDH
Fig. 4C IB: NMNAT2
Fig. 4C IB: GAPDH

Figure S7

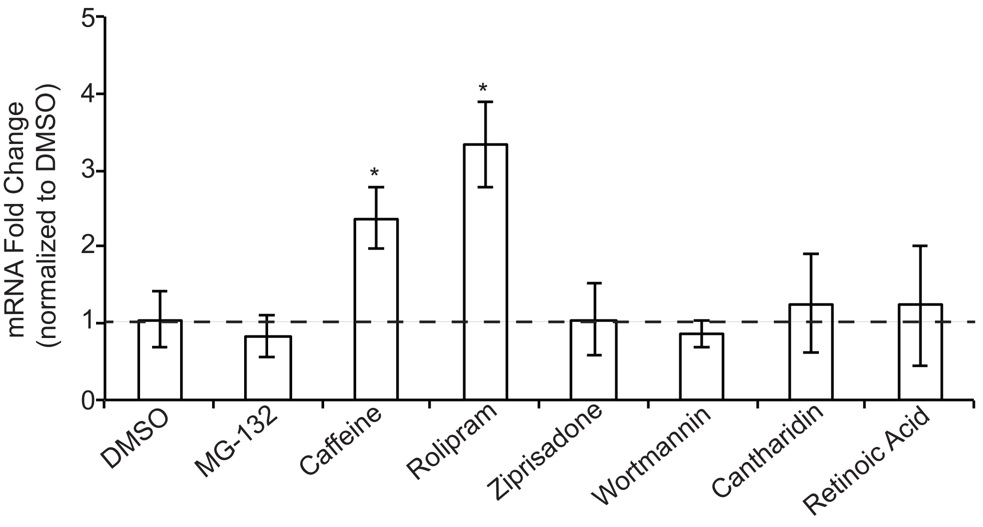
mRNA Fold Change
(normalized to DMSO)
5
4
3
2
1
0
*
*
DMSO
MG-132
Caffeine
Rolipram
Ziprisadone
Wortmannin
Cantharidin
Retinoic Acid

Figure S8
